# Encapsulated Papillary Carcinoma of Breast: Clinicopathological Features and Prognostic Parameters

**DOI:** 10.7759/cureus.11282

**Published:** 2020-10-31

**Authors:** Atif A Hashmi, Syeda N Iftikhar, Shahzeb Munawar, Arham Shah, Muhammad Irfan, Javaria Ali

**Affiliations:** 1 Pathology, Liaquat National Hospital and Medical College, Karachi, PAK; 2 Internal Medicine, Liaquat College of Medicine and Dentistry, Karachi, PAK; 3 Public Health, Baylor University, Waco, USA; 4 Internal Medicine, Ziauddin University, Karachi, PAK; 5 Statistics, Liaquat National Hospital and Medical College, Karachi, PAK

**Keywords:** encapsulated papillary carcinoma, invasive ductal carcinoma, estrogen receptor (er), progesterone receptor (pr) and human epidermal growth factor receptor-2 (her2/neu)

## Abstract

Introduction

Encapsulated papillary carcinoma (EPC) is a rare malignant papillary breast tumor that, despite a lack of distinct myoepithelial layer, is considered an in situ carcinoma unless associated with a frank invasive component. Data regarding clinicopathologic features of rare breast tumors like EPC are especially scarce. Therefore, in this study, we evaluated the clinicopathologic features of EPC and performed a clinicopathological comparison with conventional invasive ductal carcinoma (IDC).

Methods

It was a retrospective study conducted in the Department of Pathology, Liaquat National Hospital and Medical College, from January 2013 to December 2019 over a period of seven years. During this period, 16 cases were diagnosed as EPC, and 634 cases were labeled as IDC. Estrogen receptor (ER), progesterone receptor (PR), and human epidermal growth factor receptor-2 (HER2/neu) immunohistochemical (IHC) stains were performed on both EPC and IDC cases. Moreover, myoepithelial IHC stains were performed on all cases of EPC. Clinicopathologic features of EPC were compared with IDC.

Results

The mean age of the EPC patients was 51.81±13.94 years, with a mean tumor size of 2.97±2.46 cm. The majority of cases were grade II, and axillary metastasis was present in 18.8% of cases. About 56.3% of cases were in situ, and 43.8% showed foci of invasion in the form of IDC. Recurrence was noted in 12.5% of cases with a survival rate of 93.8%. ER, PR, and HER2/neu positivity was noted in 81.3%, 75%, and 12.5% cases, respectively. EPC was significantly noted to have lower tumor grade and pathological T-stage than IDC. Similarly, a lower frequency of axillary metastasis was noted in EPC than IDC.

Conclusion

EPC is a rare distinct subtype of papillary breast tumors with overall good survival and low recurrence rate. Compared to IDC, we found EPC to be associated with better prognostic parameters such as lower tumor grade and T-stage and lower frequency of axillary metastasis.

## Introduction

Papillary neoplasms are a diversified group of breast lesions that include both benign and malignant tumors [1,2]. Encapsulated papillary carcinoma (EPC) is a rare malignant papillary breast tumor that, despite a lack of distinct myoepithelial layer, is considered an in situ carcinoma unless associated with a frank invasive component. Histologically, this tumor is characterized by a thick fibrous capsule, as is thought to arise inside a single duct. Inside the fibrous capsule, the tumor is distinguished by low-grade solid proliferation of luminal epithelial cells associated with thin fibrovascular cores. The tumor lacks a myoepithelial layer in both the center of papillae and at the periphery. An invasive component that is generally in the form of conventional invasive ductal carcinoma (IDC) is sometimes associated with EPC. To be called EPC with invasion, the invasive component must be present outside the confines of the fibrous capsule. An entrapped breast epithelium along the needle tract after core biopsy sometimes mimics an invasive carcinoma causing diagnostic confusion. EPC, even without invasion, can lead to axillary metastasis in rare circumstances; therefore, most surgeons prefer a sentinel lymph node (SLN) biopsy before embarking on definite surgical resection.

EPC shows diffuse expression of hormonal receptors, i.e., estrogen receptor (ER) and progesterone receptor (PR), and lacks human epidermal growth factor receptor-2 (HER2/neu) expression. A few studies conducted to date depicted the indolent nature of this tumor [3]. Southeast Asia, especially Pakistan, is a high-risk area for breast cancer [4-6]. Owing to the lack of screening programs, patients with breast tumors typically present late, leading to a bad prognosis [7]. Moreover, young age breast tumors, including triple-negative breast tumors, are also common in this region [8,9]. Data regarding clinicopathologic features of rare breast tumors like EPC are especially scarce. Therefore, in this study, we evaluated the clinicopathologic features of EPC and performed a clinicopathological comparison with conventional IDC.

## Materials and methods

We conducted a retrospective study in the Department of Pathology, Liaquat National Hospital and Medical College, from January 2013 to December 2019 over a period of seven years. Clinicopathological data were collected from departmental archives. Cases with neoadjuvant chemotherapy were excluded from the study. During this period, 16 cases were diagnosed as EPC, and 634 cases were labeled as IDC. Specimens included lumpectomy/breast conservation surgery (BCS) or simple mastectomy with SLN biopsy and modified radical mastectomy (MRM). All patients presented in the surgical out-patient department (OPD). After clinical examination, ultrasound and mammogram were performed, followed by trucut biopsy. After a diagnosis of atypical papillary neoplasm (with or without invasion) on trucut biopsy, definite surgery was planned. For patients with clinically and radiologically negative axillary lymph nodes, SLNs were examined intra-operatively by frozen sections and, if positive (any positive SLN macrometastasis for patients undergoing MRM and more than three positive SLN for BCS patients), were followed by axillary lymph node dissection. Alternatively, patients with clinically and radiologically positive lymph nodes after confirmation by trucut biopsy/fine needle aspiration cytology (FNAC) were followed by the axillary dissection. All specimens were received in the histopathology laboratory of Liaquat National Hospital, and gross examination was done according to standard protocols. For BCS, representative radial sections were submitted from all coded (by the surgeon) resection margins along with sections from the tumor. For small tumors (<2 cm), the whole tumor was submitted. For larger tumors (>2 cm), one section per centimeter of the tumor was submitted. Additional sections were submitted if found necessary. For mastectomy, representative sections were submitted from the posterior resection margin, main tumor, skin, nipple, and areola, along with sampling from all quadrants of the breast.

**Figure 1:**
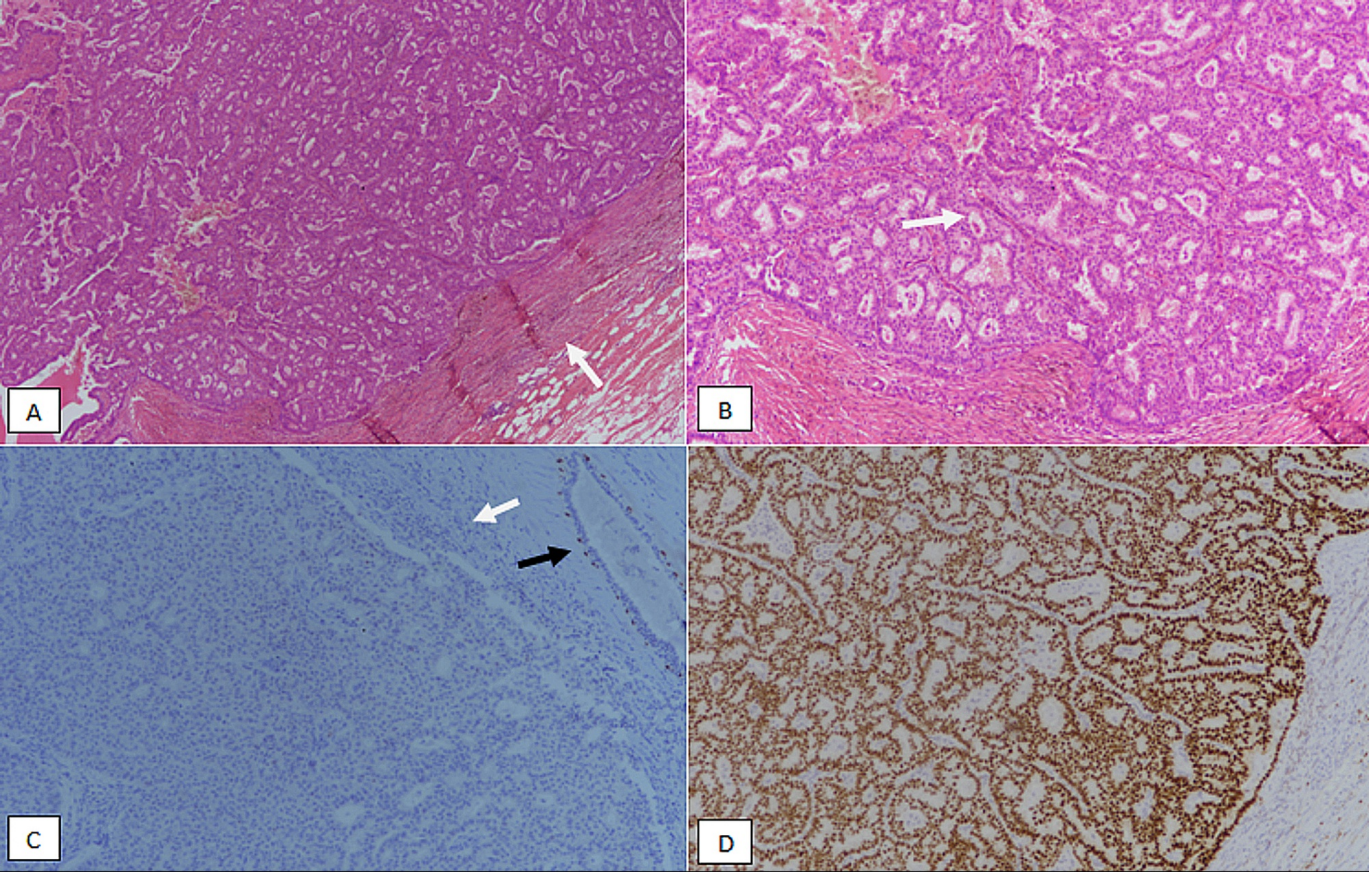
Encapsulated papillary carcinoma without invasion. (A) H&E stained sections at 100X magnification showing circumscribed papillary growth of tumor cells surrounded by a fibrous capsule (white arrow). (B) 200X magnification showing indistinct papillary cores (white arrow). (C) p63 immunostain showing a lack of nuclear myoepithelial staining at the periphery (white arrow). Intact nuclear staining is noted in normal ducts as a positive internal control (black arrow). (D) Estrogen receptor (ER) immunostain showing strong diffuse nuclear staining in tumor cells. H&E, hematoxylin and eosin

**Figure 2:**
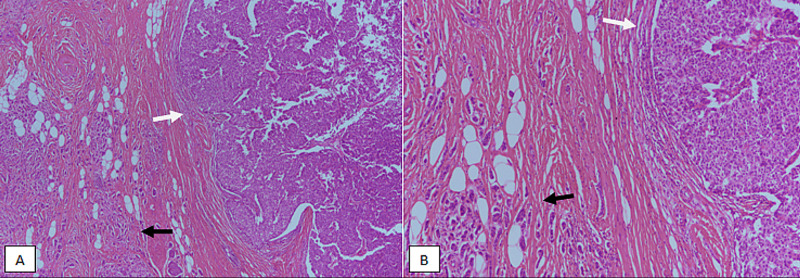
Encapsulated papillary carcinoma (EPC) with invasion, H&E stained-sections. (A) 40X magnification showing both in situ (white arrow) and invasive (black arrow) components. (B) 100X magnification showing EPC in situ with circumscribed borders (white arrow) and invasive component resembling conventional invasive ductal carcinoma (black arrow). H&E, hematoxylin and eosin; EPC, encapsulated papillary carcinoma

ER, PR, and HER2/neu immunohistochemical (IHC) stains were performed on both EPCs and IDC cases. Cases with equivocal HER2/neu IHC were accompanied by fluorescence in situ hybridization (FISH) studies to evaluate gene amplification. Moreover, myoepithelial IHC stains (p63 and myosin) were performed on all cases of EPC. Clinicopathologic features of EPC were compared with IDC. Histological images of EPC without and with the invasion are shown in Figures 1, 2.

All patients were followed up in the surgical OPD to evaluate recurrence. The clinical breast examination was performed in the OPD along with radiologic imaging. Cases with suspicious radiologic findings were followed by trucut biopsy to confirm recurrence.

Data analysis was performed using Statistical Package for Social Sciences (Version 26.0, IBM Inc., Armonk, USA). Chi-square, Fisher exact test, and independent t-test were used to check the association. P-values ≤ 0.05 were considered significant.

## Results

Clinicopathologic features of encapsulated papillary carcinoma

The mean age of the EPC patients was 51.81±13.94 years, with a mean tumor size of 2.97±2.46 cm. The majority of cases were grade II and axillary metastasis was present in 18.8% of cases. About 56.3% of cases were in situ and 43.8% showed foci of invasion in the form of IDC. Recurrence was noted in two (12.5%) cases with a survival rate of 93.8%. Two cases that observed recurrence had BCS, one of which expired due to disease. There was no additional co-morbidity present in that case. ER, PR, and HER2/neu positivity was noted in 81.3%, 75%, and 12.5% cases, respectively. The clinicopathologic features of EPC are shown in Table 1.

**Table 1:** Clinicopathologic characteristics of encapsulated papillary carcinoma SD, standard deviation; MRM, modified radical mastectomy; N, nodal; T, tumor; Tis, tumor in situ; ER, estrogen receptor; PR, progesterone receptor; HER2/neu, human epidermal growth factor receptor-2; EPC, encapsulated papillary carcinoma

Clinicopathologic characteristics	Frequency (%)
Age (years)	
Mean±SD	51.81±13.94
Age groups	
≤50 years	8 (50)
>50 years	8 (50)
Tumor size (cm)	
Mean±SD	2.97±2.46
Tumor size groups	
<2 cm	7 (43.8)
2-5 cm	7 (43.8)
>5 cm	2 (12.5)
Follow up (months) Mean±SD (Median)	23.93±17.34 (30)
Specimen type	
Lumpectomy	11 (68.8)
Simple mastectomy	1 (6.3)
MRM	4 (25)
N Stage	
N0	13 (81.2)
N1	3 (18.8)
N2	0 (0)
N3	0 (0)
T Stage	
Tis	9 (56.3)
T1	7 (43.8)
T2	0 (0)
T3	0 (0)
Grade	
Grade-I	3 (18.8)
Grade-II	12 (75)
Grade-III	1 (6.3)
Lymphovascular invasion	
Present	0 (0)
Absent	16 (100)
Axillary metastasis	
Present	3 (18.8)
Absent	13 (81.2)
ER	
Positive	13 (81.3)
Negative	3 (18.7)
PR	
Positive	12 (75)
Negative	4 (25)
HER2/neu	
Positive	2 (12.5)
Negative	14 (87.5)
Type of EPC	
EPC in situ	9 (56.3)
EPC with invasion	7 (43.8)
Recurrence	
Yes	2 (12.5)
No	14 (87.5)
Survival Status	
Alive	15 (93.8)
Expired	1 (6.2)

The most common clinical presentation was breast lump (93.75%), followed by nipple discharge (18.75%). Radiological findings were available in 13 cases. The hypoechoic solid mass was the most common ultrasound findings, with breast imaging-reporting and data system (BI-RADS) 4 being the most common BI-RADS assessment category on mammograms. Detailed radiological and clinical findings are presented in Table 2.

**Table 2:** Clinical and radiological findings of patients with encapsulated papillary carcinoma BI-RADS, Breast imaging-reporting and data system

Clinical/ radiologic finding	Frequency (%)
Clinical presentation (n=16)	
Breast lump	15 (93.75)
Breast pain	1 (6.25)
Nipple discharge	3 (18.75)
Nipple retraction	1 (6.25)
Ultrasound findings (n=13)	
Hypoechoic solid mass	8 (61.5)
Complex cyst	3 (23.07)
Mural based nodule	1 (7.69)
Mamogram findings (n=13)	
BI-RADS assessment category	
BI-RADS 0	1 (7.69)
BI-RADS 3	1 (7.69)
BI-RADS 4	7 (53.84)
BI-RADS 5	4 (30.76)
Margins	
Well defined	9 (69.23)
Indistinct	3 (23.07)
Spiculated	1 (7.69)
Microcalcifications	
Present	3 (23.07)
Absent	10 (76.92)

Comparison of encapsulated papillary carcinoma with invasive ductal carcinoma

Table 3 shows the comparison of clinicopathological features of EPCs with IDC. EPC was noted to have significantly lower tumor grade and pathological T-stage than IDC. Similarly, a lower frequency of axillary metastasis was noted in EPC than IDC. EPC was found to have a higher frequency of ER and PR positivity and lower HER2/neu positivity; however, the results were not statistically significant.

**Table 3:** Comparison of encapsulated papillary carcinoma with invasive ductal carcinoma of the breast *Chi-square test was applied, **Fisher Exact test was applied, ***Independent t-test was applied. SD, standard deviation; N, nodal; T, tumor; Tis, tumor in situ; ER, estrogen receptor; PR, progesterone receptor; HER2/neu, human epidermal growth factor receptor-2

Clinicopathologic characteristics	Encapsulated papillary carcinoma (n=16)	Invasive ductal carcinoma (n=634)	P-value
Age (years)			
Mean±SD	51.81±13.94	51.95±12.15	0.965***
Age groups			
≤50 years	8 (50)	306 (48.3)	0.891**
>50 years	8 (50)	328 (51.7)	
Tumor size (cm)			
Mean±SD	2.97±2.46	3.61±1.48	0.098***
Tumor size groups			
<2 cm	7 (43.8)	52 (8.2)	0.0001**
2-5 cm	7 (43.8)	485 (76.5)	
>5 cm	2 (12.5)	97 (15.3)	
N Stage			
N0	13 (81.2)	320 (50.5)	0.053**
N1	3 (18.8)	130 (20.5)	
N2	0 (0)	85 (13.4)	
N3	0 (0)	99 (15.6)	
T Stage			
Tis	9 (56.3)	0 (0)	0.0001**
T1	7 (43.8)	83 (13.1)	
T2	0 (0)	454 (71.6)	
T3	0 (0)	97 (15.3)	
Grade			
Grade-I	3 (18.8)	53 (8.4)	0.002**
Grade-II	12 (75)	293 (46.2)	
Grade-III	1 (6.3)	288 (45.4)	
Lymphovascular invasion			
Present	0 (0)	157 (24.8)	0.017**
Absent	16 (100)	477 (75.2)	
Axillary metastasis			
Present	3 (18.8)	314 (49.5)	0.015*
Absent	13 (81.2)	320 (50.5)	
ER			
Positive	13 (81.3)	399 (62.9)	0.133*
Negative	3 (18.7)	235 (37.1)	
PR			
Positive	12 (75)	323 (50.9)	0.057*
Negative	4 (25)	311 (49.1)	
HER2/neu			
Positive	2 (12.5)	223 (35.2)	0.060*
Negative	14 (87.5)	411 (64.8)	

## Discussion

In this study, we evaluated the clinicopathological features of a rare breast cancer subtype, i.e., EPC, and compared its clinicopathological features with IDC. We noted that in our study, EPC had lower tumor grade and T-stage than IDC. Moreover, axillary metastasis, an important prognostic factor in breast cancer, was lower in EPC compared to IDC.

EPC is considered an indolent variant of breast cancer. A clinicopathologic study of 49 cases of EPC revealed hormone receptor expression in more than 90% of cases and nodal metastasis in 7.7% cases. Follow-up revealed local recurrence in 17% of cases [10]. In our study, ER expression was seen in 81.3% cases and nodal metastasis was noted in 18.8% cases that were lower than IDC (49.5%).

EPC is a biologically unique category of breast carcinoma with an invasive potential intermediate between ductal carcinoma in situ (DCIS) and IDC of breast. A study assessed the invasive potential of EPCs by evaluating the expression of matrix metalloproteinases, transforming growth factor receptor-beta, vascular endothelial growth factor (VEGF), and E-cadherin in EPC and concluded that EPC exhibited expression of invasion-associated markers, but to a lesser degree compared to IDC of the breast [11]. Despite this invasive potential, EPC is staged as in situ carcinoma unless associated with an invasive component outside the tumor capsule. In our study, invasive carcinoma associated with EPC was noted in 43.8% of cases.

EPC is a diagnostic challenge on core needle biopsy, and thus, understanding the role of myoepithelial markers is pivotal. Any papillary tumor with a lack of myoepithelial staining at the periphery of the tumor should be diagnosed as atypical (if there is no evidence of invasive component) and advised for excision. However, it is difficult to assess the periphery of the tumor on core needle biopsy for the evaluation of myoepithelial markers. In our study, most of the cases were diagnosed as atypical papillary neoplasm on core needle biopsy, and surgical excision was advised.

Racha et al. evaluated 208 cases of intracystic papillary carcinoma (old terminology for EPC) and observed only 3% incidence of nodal metastasis and infrequent development of local or distant recurrence, thus conferring excellent prognosis [12]. We found local recurrence in two cases, both of which had BCS with the closest 2-mm resection margin. Additionally, both of these cases had an invasive component. The presence of invasion is an important factor determining disease outcome in cases with EPC. A study conducted in Pakistan evaluated 25 cases of EPC and confirmed invasion in 44% cases [13]. We found invasion in 43.8% cases. Despite the presence of invasion, overall pathological parameters were better in EPC than IDC in our study.

We acknowledge a few limitations of our study. First, the number of cases was few. Second, the survival status of IDC was not evaluated for comparison with IDC that should be addressed in future studies of the same nature.

## Conclusions

EPC is a rare indolent subtype of breast cancer with good overall survival. Compared to IDC, EPC was found to have better prognostic pathological parameters such as lower grade and T-stage and lesser frequency of axillary metastasis. Understanding the histological features of this rare papillary breast tumor is essential for pathologists, especially identifying the presence of invasion as we found invasion in a substantial number of EPC cases in our study.
